# Diagnostics of atherosclerosis: Overview of the existing methods

**DOI:** 10.3389/fcvm.2023.1134097

**Published:** 2023-05-09

**Authors:** Anastasia V. Poznyak, Vasily N. Sukhorukov, Ilya I. Eremin, Irina I. Nadelyaeva, Alexander N. Orekhov

**Affiliations:** Institute of General Pathology and Pathophysiology, Russian Academy of Medical Sciences, Moscow, Russia

**Keywords:** atherosclerosis, cvd, cardiovascular disease, diagnostics, diagnostic tools

## Abstract

Atherosclerosis was and remains an extremely common and serious health problem. Since the elderly are most at risk of cardiovascular risk, and the average life expectancy is increasing, the spread of atherosclerosis and its consequences increases as well. One of the features of atherosclerosis is its asymptomaticity. This factor makes it difficult to make a timely diagnosis. This entails the lack of timely treatment and even prevention. To date, in the arsenal of physicians, there is only a limited set of methods to suspect and fully diagnose atherosclerosis. In this review, we have tried to briefly describe the most common and effective methods for diagnosing atherosclerosis.

## 1. Atherosclerosis

In the 19th century, atherosclerosis was characterized as a disease associated with the accumulation of lipids, mainly cholesterol, in the arteries. Later, in the middle 20th century, it became clear that atherosclerosis is treatable. Studies that have been conducted on animals and in individual patients have shown that with manipulations that reduce circulating levels of lipoproteins containing cholesterol, atherosclerosis was reduced, and clogged arteries partially opened (1).

Recently, strong cholesterol-lowering drugs, as well as the development of improved non-invasive methods for assessing vascular diseases, have confirmed that atherosclerosis can be both reduced and cured. To determine the mechanisms of this, an animal model was first needed, which would have high levels of circulating cholesterol and atherosclerotic lesions. Rats do not develop high cholesterol levels, even when their dietary cholesterol is significantly elevated; this is because their liver reduces its cholesterol biosynthesis (2, 3).

On the contrary, rabbits fed with cholesterol develop atherosclerosis, partly due to the relative deficiency of hepatic lipase, the final enzyme in the metabolism of chylomicron, and very low-density lipoproteins (VLDL) metabolism. For the first time, regression in this model was noted when researchers demonstrated that a return to the usual rabbit diet leads to a decrease in the number of cholesterol-rich arterial plaques (4). Studies on monkeys and pigs confirmed bidirectional changes in the size of atherosclerotic plaques associated with changes in cholesterol levels in the blood (5).

Studies on rabbits have also shown that the size and/or composition of lipoproteins plays a key role in the development of atherosclerosis. This was revealed by chance during a study of the relationship between atherosclerosis and diabetes; in rabbits with diabetes, the disease decreased, although the levels of circulating cholesterol and triglycerides were elevated (6). The reason was that the circulating lipoproteins, mainly chylomicrons, were too large to get into the artery wall.

Initially, hamsters were used as model animals for atherosclerosis. After feeding with the high-cholesterol diet, golden Syrian hamsters developed scattered but advanced lesions mimicking the human condition (7). The metabolism of lipids in hamsters, including their synthesis, processing, binding to ligands, and recycling, is more similar to that of humans than in rats and mice. However, variable susceptibility to an atherogenic diet, as well as multiple measurements of aortic lesion development, also made it more difficult to compare morphologic changes between studies (8).

Mice can be genetically altered to lack apolipoprotein (Apo)E, which is required for clearance of partially metabolized (remnant) lipoproteins, to lack the low-density lipoprotein receptor (LDLr), or to overexpress ApoB (9). These mice develop hypercholesterolemia and atherosclerosis, especially when they are fed a diet containing a large amount of cholesterol and saturated fats. These single genetic variations are enough to provoke atherosclerosis in animals that are quite resistant to atherosclerosis. Therefore, the only ingredient necessary for the formation of atherosclerotic lesions is an increased level of ApoB lipoproteins (10).

Over the past ten years, various techniques have emerged to investigate the regression of atherosclerosis in mice. In some models, switching from a high-cholesterol diet to a normal chow diet leads to regression of atherosclerosis, usually when blood cholesterol levels are reduced to below 200 mg/dl. The transplantation of aortic segments with lesions generated from hypercholesterolemia to mice with normal cholesterol levels results in the regression of the lesions (11). Another regression method involves the genetic reversal of hypercholesterolemia. These experiments made it possible to identify many biological processes involved in normal and defective regression (12).

## 2. Diagnostics

Until nowadays, the diagnosis of atherosclerosis has been carried out by such methods as evaluation of electrocardiogram (ECG) both at rest and during physical activity, evaluation of the Ankle Brachial Pressure Index (ABPI), and invasive angiography (13). Currently, the visualization of plaques is possible with the help of non-invasive imaging methods. The clinical practice employs several methods for plaque imaging, including ultrasound, magnetic resonance tomography (MRI), computer tomography (CT), positron emission tomography (PET), and single-photon emission computed tomography (SPECT) (nuclear imaging techniques) (14). Liposomes can serve as diagnostic agents for the noninvasive early detection of atherosclerosis, in addition to the imaging methods previously mentioned. Liposomal imaging agents transmit signals straightaway from the site of a lesion, which makes it possible to locate the plaque, to identify its size and structure. In the diagnosis of atherosclerosis, liposomes are extremely important, since they are responsible for the transfer and delivery of contrast agents, which enhances the quality of image resolution. By loading several contrast agents, liposomes are able to become multifunctional (15).

Visualization methods have a variety; a specific method is selected based on the stage of plaque development. For example, endothelial dysfunction at an early stage can be diagnosed by functional measurements such as peripheral arterial tonometry (PAT) and also can be visualized by PET and CT. Using coronary intravascular ultrasound, MRI, and coronary computed tomography, more progressive lesions with lipid buildup can be found. Expanded plaques can be found using electron beam computed tomography (16, 17). Imagining atherosclerosis includes a set of both proven and test radiological techniques and modalities. In general, these methods may be applied to identify the anatomical and physiological impact of prolonged atherosclerosis, to obtain detailed information about the composition of plaques and molecular activity, as well as to assess biomechanical stresses acting in the arterial system. Together, these methods provide measures of the severity of the disease, which are necessary for everyday clinical practice and cardiovascular (CV) research (18).

## 3. Structural and functional imaging of atherosclerosis

**Figure 1:**
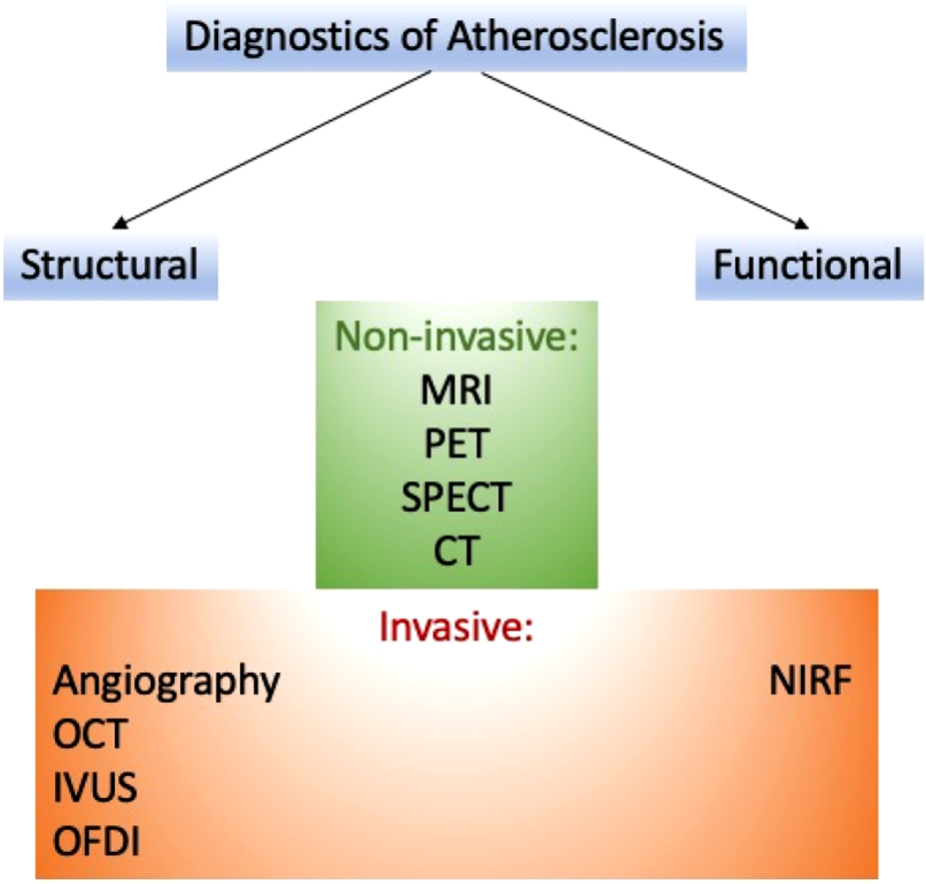
The variety of methods in diagnostics of atherosclerosis.

Several approaches can help detect atherosclerosis. These methods can be classified on various bases (see Figure 1).

### 3.1. Structural imaging

To visualize the vascular structure of atherosclerosis, numerous imaging methods are used, including the measurement of the volume of the lesion and the thickness of the fibrous membrane. The widely used methods are magnetic resonance imaging (MRI) and magnetic resonance angiography (MRA), which uses gadolinium chelates/nanoparticles (Gd), superparamagnetic iron oxide probes (SPIO), ultra-small superparamagnetic iron oxide (USPIO) as contrast enhancement with a resolution of 10–100 microns to imagine the structure of atherosclerotic lesions (19).

Another method is computed tomography (CT), in which iodine-containing molecules as imaging moieties and high-resolution x-rays are used as a technology with a resolution of 50 µm for clinical or preclinical imaging (20). Methods such as positron emission tomography (PET) and single-photon emission computed tomography (SPECT) are gaining popularity because they use imaging components such as ^18^F, ^64^Cu, ^11^C Tracers/^99m^Tc, ^123/124/125/131^I, ^111^In tracers and nuclear technology with a resolution of ∼2 µm (21). Widely used invasive approaches for the detection of atherosclerotic lesions are (1) angiography (fluoroscopy using iodine-containing molecules as a contrast agent); (2) optical coherence tomography (OCT); (3) optical imaging in the frequency domain (OFDI), optical angioscopy; (4) and intravascular ultrasound (22).

### 3.2. Functional imaging

Visualization of specific cells or components within a lesion is able to reveal the biology and characteristics of the lesion, including its susceptibility to serious CV complications. This information can be crucial in preventing such complications. By including peptides, antibodies, or other ligands on its surface, the nanoparticle can be targeted at the components of the lesion (i.e., collagen, proteinases, reactive oxygen species) and cells.

Diagnostic agents or contrast agents incorporated into nanoparticles can be found using imaging methods such as MRI, PET/SPECT, CT, and near-infrared fluorescence (NIRF). Fluorescent imaging cannot be used in clinical studies due to low penetration. However, it is a decent approach to the image of atherosclerosis in small animal models. An alternative approach is to use nanoparticles that are conjugated with specific ligands that target adhesion molecules, allowing for the visualization of dysfunctional endothelial cells. One of the most common inflammatory cells in atherosclerosis are macrophages and foam cells. They have phagocytic activity, express scavenger receptors (i.e., CD 36, LOX-1, MSR1), and also secrete reactive oxygen species (oxidized epitopes) and matrix-destroying proteases (i.e., matrix metalloproteinases and cathepsins). Together, these signs can be potential targets for visualizing macrophages and foam cells and evaluating their oxidative and inflammatory activity (23, 24).

Fibrin and factor XIII can be used to target thrombosis. Integrin avß3 can be used to visualize neoangiogenesis of the lesion. The large number and distribution of these cells, as well as the main active components in the lesions, provide valuable information beyond the scope of the lesion. Events such as inflammation, especially neoangiogenesis, destruction of the fibrous membrane, and oxidative stress are crucial for the choice of both preventive and therapeutic methods (25).

### 3.3. PET & SPECT: nuclear imaging

Nuclear imaging techniques, including PET and SPECT, rely on a source of radiation within the body. These methods utilize a small amount of radioactive tracer that is administered to the patient. The tracer releases gamma rays, which are then captured by specialized cameras to produce images. These images reveal the distribution and concentration of the tracer in the body and provide valuable information about the biological processes being studied. FDG is a radioactively labeled glucose equivalent that may be helpful in PEТ. As for atherosclerosis, this method is excellent for detecting macrophages and inflammation (26). Another PET reagent used for the dynamic evaluation of coronary microcalcification is 18F-sodium fluoride. Also, a SPECT with various indicators can be used to establish inflammation (27, 28). The liposome-based process is currently being investigated to improve both PET and SPECT imaging in various diagnostic conditions. For instance, PS-containing liposomes of 100 or 200 nm (PS100 and PS200) were injected into hereditary hyperlipidemic Watanabe rabbits, scanned using SPECT, and compared with CT images 48 h after injection (15, 26–28).

### 3.4. CT

Computed tomography is fast and relatively inexpensive. For the successful application of this technique, a bolus injection of a contrast agent is required. Computed tomography is suitable for detecting calcification in atherosclerosis. In CT, liposomes play the same role as in MRI. Liposomes (simple or pegylated), as a rule, contain a contrast agent for computed tomography, e.g., iopromide, gold, or bismuth (29). Danila and colleagues developed a method to mitigate the negative effects of free iohexol, including its short presence and renal toxicity, by encapsulating a contrast agent within PEGylated liposomes. The liposomes were composed of DPPC, cholesterol, and a linker in a 3:1:0.3 molar ratio. They were subsequently connected with an anti-ICAM-1 antibody for targeting the plaque (30).

### 3.5. MRI

MRI is beneficial for the determination of diverse elements of plaque; the fibrous membrane and the lipid layer are among them. It provides detailed information about the plaque, such as its size, composition, endothelial permeability, and neovascularization. Additionally, MRI offers high-resolution 3D images that approach the level of cellular detail. This makes it ideal for detecting macrophages and macrophage-rich areas, as well (31). Targeting liposomes to the plaque stimulates the accumulation of the loaded contrast agent at the plaque site, which leads to an improvement in the signal level. PEGylated liposomes containing Gd-DTPA are liposomes that are formulated with an MRI contrast agent. Furthermore, as previously mentioned, liposomes that are enriched with phosphatidylserine (PS) are detected by macrophages within plaques. The presence of gadolinium in liposomes can improve the buildup of contrast agents at the site of plaques, leading to more accurate detection of the target area. The storage of gadolinium-loaded liposomes within plaque is linked to the endoplasmic reticulum retention receptor (ERR). Additionally, liposomes functionalized with antibodies directed towards the LOX-1 receptors in the dysfunctional endothelium of the plaque have been used to load gadolinium (32).

There have been other instances where liposomes with gadolinium have been used in MRI. For example, Paulis et al. utilized Gd-DOTA and DSPE to improve the targeting of ICAM-1 (33). Another study used the MARI — integrin antagonist a4b1 (THI0567) — was added into liposomes, which contains Gd. The liposomal drug binds to integrin *α*4*β*1 receptors on monocytes and has been used to locate plaques in the aortas of ApoE^−/−^ mice, which are known to be susceptible to atherosclerosis (34). Thus, target liposomes with a gadolinium-based contrast agent ensure low background amplification and are fit for magnetic resonance imaging of endothelial markers that present in large quantities in plaque deposits.

### 3.6. Ultrasonography

Ultrasound imaging techniques help to find vulnerable atherosclerotic plaques. In virtue of ultrasound, it becomes an option to carry out catheter-based measurement of the thickness of the intima-media of the carotid artery in real time (35, 36). There are also brand-new methods that can give more accurate data on the morphology of the arterial wall, for example, such a method as intravascular photoacoustic-ultrasound (IVPA-US) imaging. The superiority of intravascular photoacoustic ultrasound over ordinary ultrasound techniques is that it can deliver data on the plaque structure.

The liposome has a layered structure, which makes it possible to catch gas bubbles that can efficiently mirror sound waves and produce acoustically reflective liposomes. These liposomes can be conjugated with antibodies, such as anti-fibrinogen or anti-ICAM-1, to enhance plaques' recognition and targeting (37). Multilayer acoustic liposomes containing PC, PE, PG, and cholesterol were obtained with trapped bubbles of gas among the lipid layers. This led to a large acoustic increase during sonography when evaluating induced atherosclerosis in Yucatan mini-pigs (15). Another method is the injection of a contrast agent like IJA into liposomes. The liposomes are directed towards FRb, which is highly expressed on macrophages within atheromatous plaques. In an ApoE^–/–^ mouse model with atherosclerosis, the higher absorption of FRb-targeted liposomes was observed *in vivo* (38).

## 4. Imaging plaque morphology and composition

In addition to the usual anatomical and hemodynamic assessments of the severity of the illusion, when visualizing the vessel wall, a detailed characterization of the plaque can be obtained. The information obtained through autopsy studies conducted among patients with coronary artery disease (CAD) whose death was unexpected provides a histopathological foundation for identifying high-risk plaques and helps visualization of highly sensitive plaques (39, 40). The results of the studies made it clear that, as a rule, the underlying plaque morphology resulting in a MI includes a slim torn fibrous cap with strong penetration by macrophages and a few SMCs, a vast necrotic nucleus, and overlying intraluminal thrombosis. Intima neovascularization is a reason for intra-plasma hemorrhage, which provokes a very high risk of plaque rupture. Erosion of a plaque occurs in 30%–35% of cases of sudden coronary death, and blood clots associated with calcified nodes occur in 2%–7% of cases (41).

The thin-cap (<65 μm) fibroatheroma (TCFA) has a close resemblance to a ruptured plaque, however, does no longer have lumen thrombosis and is consequently considered because of the maximum probable lesion preceding the rupture of the plaque (42). In fact, cap thickness is the high-quality histological indicator of a coronary plaque kind, accompanied by infiltration by macrophages and a necrotic nucleus. The histopathological appearance of currently appeared symptomatic carotid plaques resembles the culpable lesions of the coronary arteries, although the thickness of the cap seems less important. Because the majority of ruptured coronary plaques arise in a limited, focal distribution, grouping in particular in the proximal coronary vascular network, both invasive and non-invasive characteristics of plaques are allowed. Thin-cap, big necrotic nucleus, positive remodeling microcalcification, and neovascularization are a number of the recognized symptoms of excessive-threat coronary plaques detected *in vivo*; regardless of this, the significance of excessive-threat plaque imaging in recurring clinical practice remains to be decided (43).

## 5. Intravascular coronary imaging (IVUS)

Intravascular imaging techniques such as IVUS, OCT, and NIRS are able to provide extensive data about the composition of coronary plaques in patients undergoing invasive angiography. The IVUS catheter uses sound waves in the frequency range of 20–60 MHz, generated by the rapid oscillation of a piezoelectric transducer (44). While traditional gray-scale intravascular ultrasound has limited ability to distinguish between plaque components, the use of spectral analysis techniques such as Virtual Histology (VH)-IVUS can accurately identify necrotic core, dense calcium, fibrous plaque, and fibrous-fat plaque based on radio frequency backscattering data.

Due to increased noise and artifacts, interpretation of the image may be difficult. Moreover, the IVUS has an insufficient spatial resolution for reliable and reproducible detection of a thin fibrous cap (45). OCT uses near-infrared light (wavelength 1.3 μm) emitted via a fiber optic cord with a rotating lens to achieve rather excessive spatial resolution (10–15 μm). Recent studies have demonstrated that Optical Coherence Tomography (OCT) is a highly accurate method for evaluating the thickness of the fibrous cap in atherosclerotic plaques. OCT measurements show a strong correlation with histology, and their sensitivity and specificity for identifying plaque types are excellent. However, distinguishing between calcium and lipid pools in plaques can be difficult with OCT due to limited tissue penetration (up to 3 mm), making it challenging to estimate the entire plaque volume. To obtain bloodless images, saline or contrast is injected during the pullback process. OCT also holds promise for other applications, including elastography, polarization-sensitive OCT (PS-OCT), and OCT Doppler, in addition to its diagnostic capabilities.

OCT's high resolution enables it to provide information about plaque composition at the cellular level. In an ex vivo study, OCT was used to quantify macrophages within the fibrous cap. These were visible as vibrant spots with higher signal depth than encompassing structures and an unexpected drop in OCT signal (46). Despite the interesting discovery, further studies have shown that only 23% of areas with positive light spots on OCT mostly represent only macrophages.

Another ex vivo study in human coronary arteries using micro-OCT (spatial resolution of 1 μm) showed incredible images of cellular and subcellular structures, including leukocytes bound to the surface of the endothelium, similar in appearance to electron microscopy. To identify TCFA, VH-IVUS, and OCT have similar diagnostic accuracy (76%–79%), which can be further improved by the introduction of hybrid imaging catheters (47, 48). NIRS uses scattered reflecting near-infrared light (wavelength 0.8–2.5 μm) to create a chemogram of vessel wall components based on the detection of various uptake and scattering patterns. Even though NIRS can determine the content of lipids underlying excessive-threat plaques in human arteries via blood, its key limitation is the absence of any structural information about the plaque. One approach to addressing the limitations of OCT in estimating the thickness of the fibrous cap in atherosclerotic plaques is through the use of hybrid imaging, specifically NIRS-IVUS (47). However, despite the benefits of this hybrid catheter, it is still unable to provide reliable estimates of cap thickness.

## 6. CT-Derived plaque morphology

Along with identifying the anatomy of the coronary arteries and the severity of lumen stenosis, coronary computed tomography angiography can help with data on the morphology and composition of plaques. The thickness of the fibrous cap and the necrotic nucleus is considered the key histological predictors of plaque rupture. Even though the spatial resolution of coronary computed tomography angiography is not enough to measure the thickness of the cap, it can still detect necrotic nuclei and CFAs, which are typically large enough to be visible on CT images. Coronary computed tomography angiography can also categorize plaques into calcified, partially calcified (<50%), or non-calcified types. However, when assessing plaque size, coronary computed tomography angiography tends to downplay the size of uncalcified plaques and exaggerate the size of calcified plaques due to the blooming artifact. In comparison to intravascular ultrasound, coronary computed tomography angiography has an approximately 90% susceptibility for detecting non-calcified plaques with intima thickness >1 mm (49).

Fibrous plaques appear brighter on CT scans and have less brightness around the necrotic nucleus and fibrous-adipose tissue when compared to VH-IVUS. An excellent correlation was also shown between the signs of high-risk CT and TCFA on OCT and the burden on the plaques induced by CT on the deposition of cholesterol in NIRS. According to IVUS studies, a Hounsfield unit <30 per coronary computed tomography angiography was considered as a criterion for detecting lipid-rich plaques, from 30 to 150 Hounsfield units for fibrous and >220 Hounsfield units for calcified (50).

Nonetheless, the use of the absolute value of CT attenuation to determine the composition of the plaque is a difficult task because it is necessary to take into account the influence of various factors. These factors are (1) the size of the necrotic nucleus, (2) wall thickness, (3) the measurement point, (4) intraluminal contrast density, (5) slice thickness, and (6) the reconstruction filter. The attenuation range with contrast adjustment is theoretically able to enhance the accuracy of the evaluation of the components of the coronary computed tomography angiography plaque. Unstable lesions displayed by coronary computed tomography angiography in people with ACS are usually not calcified in individuals with chronic stable angina, and larger plaques with spotty calcification and a higher remodeling index are found to have reduced brightness, compared to solid lesions. This is observed in both men and women (51, 52). Positive (external) remodeling happens due to compensatory expansion of the vessel wall, which results in the formation of large-volume plaques with often a slight narrowing of the lumen. A trait associated with a large lipid nucleus and a high number of macrophages. The limit of positive remodeling on CT is the cross-sectional area >10% of the adjacent control segment, and spot calcification is defined as <3 mm in all directions (51–54, 53).

Spotted calcification reflects small deposits of calcium in the plaque structure instead of genuine microcalcification, which may be a reaction to inflammation and destabilizes the plaque, affecting the local stress concentration. Upon detection by intravenous infusion, spot calcification is linked with diffuse atherosclerosis and speeds up the development of the disease. The sign of the CT napkin ring indicates a region of reduced density near the interior of the vessel, distinguished by a surrounding ring of increased density (55). This feature suggests the presence of a necrotic nucleus enriched with lipids and fibrous components of TCFA. Plaques that are characterized by low attenuation, positive remodeling, and a sign of a napkin ring on CT are predictive signs linked with an elevated hazard of myocardial infarction occurrence. It is noteworthy that these signs of high-risk plaques are also 3–5 times more common in FFR-positive lesions than in non-obstructive diseases (56).

## 7. MR-Derived plaque morphology

Analysis of coronary plaques using MRI is not so beneficial for clinical purposes compared with CCTA; on the other hand, it is possible to measure the wall thickness of proximal vessels using this method. Using black-blood MRI in asymptomatic people with CV risk factors, positive remodeling and an elevation in the thickness of the coronary wall was demonstrated. Information was also provided indicating the visualization of wall edema associated with lesions caused by ACS using MRI with a short inversion recovery sequence weighted by T2 (57, 58). Moreover, a hyperintense coronary signal on an MRI with T1-weighing can be a marker of high-risk plaques, which is associated with the severity of clinical angina and increased CV risk. Probably, the hyperintense T1 plaque signal is triggered by the formation of methemoglobin while a subclinical plaque rupture or hemorrhage; this is the best present-day MRI method for detecting high-risk coronary plaques (59).

The carotid arteries are relatively immobile (unlike coronary imaging) and have the sufficient caliber to be able to examine the morphology of plaques using multi-contrast weighted MRI or intravenous gadolinium-based contrast. Sequences T1 and T2 on a multi-contrast weighted MRI differentiate the elements of the plaque, which exhibit different relaxation properties and signal strength (60). This method allows accurate measurement of the thickness of the carotid artery wall without intravenous contrast on a standard 1.5-T scanner. On the other hand, it has been shown that using MRI with a higher field strength of 3.0-T, it is possible to obtain higher image quality due to an advanced signal/contrast/noise ratio.

On an MRI, fibrous tissue exhibits a low signal on T1 and a high signal on T2-weighted scans. On the other hand, calcium appears hypodense on both types of scans. Using high-quality 3D MRI, the fibrous cap is shown as a low signal juxta luminal band that is nonexistent in the presence of a thin or torn cap (61). The rupture of the cap, detected by MRI, was shown in connection with the recently appeared symptoms of carotid plaques. Intraplaque hemorrhage leading to a high-intensity T1 signal in the carotid arteries correlates with an elevation in the level of C-reactive protein, symptomatic rupture of the cap, and an elevated hazard of stroke in the future (62).

Gadolinium-based contrast MRI may be applied to quantify the size of both the fibrous cap and necrotic nucleus saturated with lipids. In T1-weighted images after intravenous contrast, the lipid core shows less gain compared to the surrounding fibrous tissue. Besides, elevated lipid core on MRI is an essential prognostic marker. Dynamic contrast amplification has been used for MRI imaging of the carotid arteries. Using this method, dynamic images obtained before and after the introduction of gadolinium contrast are assessed using kinetic modeling to derive transfer constant (Ktrans), which is associated with the content of macrophages in the carotid plaque and neovascularization (63).

## 8. Molecular imaging

The mechanisms underlying the atherosclerosis pathophysiology and its medical results can be illuminated using relatively vulnerable probes for molecular imaging. Non-invasive molecular imaging platforms based on nuclear tomography and MRI were thoroughly studied mainly *in vivo*, while molecular CT, ultrasound, and NIRF are among other novel research methods (64, 65). Unlike other methods, including SPECT and MRI, PET has excellent sensitivity for detecting molecular signals even at picomolar concentrations in tissues, which makes this method more advantageous compared to others. Nevertheless, the limited spatial resolution (4–5 mm) indicates that images are required to be recorded using either CT or MRI for accurate anatomical localization of the PET signal. Molecular probes being applied for the visualization of atherosclerosis may be additionally applied to quantify vascular inflammatory process, early calcification, plaque hypoxia, and neoangiogenesis (66).

The most widely used technique is PET with 18F-fluorodeoxyglucose (18F-FDG) or 18F-sodium fluoride (18F-NaF) as radioactive tracers. Indicator uptake is usually calculated as Standardized Uptake Values (SUVs), describing the ratio of activity per unit volume of the area of interest (ROI) to activity per unit volume of the entire body (8). Tracer uptake is calculated as a maximum value (SUVmax), which represents the maximum absorption in one voxel, or an average value (SUVmean), which averages all absorption in the region of interest. SUVmean has an advantage over SUVmax because SUVmax can be easily affected by background uptake of the radiopharmaceutical by adjacent tissues or motion artifacts. In addition, focusing only on the area with the highest avidity of the radiopharmaceutical, as is done with SUVmax, conceal the complex origin of the disease mechanisms (67).

18F-FDG is a radioactively labeled analog of glucose. It notes inflammation and metabolic activity. The highest uptake of 18F-FDG was observed in macrophages (68). An investigation conducted by Ogawa et al. showed that 18F-FDG accumulation increases during foam cell development (69). However, the uptake reaches the control level after macrophages completely differentiate into foam cells. Such result contributes to the detection of the early stage of foam cell formation in atherogenesis by 18F-FDG PET. Moreover, an association between 18F-FDG uptake and hexokinase activity was also shown. In addition, macrophages are involved in the plaque inflammation, while ruptured plaques have been shown to include numerous macrophages. Therefore, the eficacy of 18F-FDG in assessing plaque vulnerability is supported by the observation that 18F-FDG uptake is directly proportional to macrophage density (70). Because inflammatory cells needs higher amounts of glucose, unlike other cells presented within the plaque, 18F-FDG PET imaging can highlight inflammatory activity in atherogenesis, presumably helping to detect unstable plaques (71).

18F-NaF is commonly used as a marker of bone mineralization in skeletal imaging. The mechanism of the indicator includes an exchange of hydroxyl groups, and during this process 18F- ion is introduced into an open hydroxyapatite crystal (72). Due to this, it helps indicating the calcification and ossification regions, and can detect primary and metastatic bone disease. Since the atherogenesis involves calcification, the action of 18F-NaF is considered a potential indicator for atherosclerosis detecting (73). Research by Creager and colleagues has shown the highest affinity of 18F-NaF has a to hydroxyapatite, that exceeded the affinity to other important calcium salts (74). The microcalcifications with the greater surface area have the better absorption of 18F-NaF ligand binding to the surface of the calcification. However, for macrocalcifications, the radioactivity signal is much weaker. Thus, the specificity of 18F-NaF for microcalcification introduces the use of the indicator to detect atherosclerotic plaques at an early stage.

Macrocalcifications found on CT are considered stable areas where the atherosclerotic disease is at rest, while areas with 18F-NaF uptake show active microcalcifications, which are considered to be unstable plaques. The investigation of atherosclerotic plaques by molecular imaging mediated not only by 18F-NaF and 18F-FDG (75). Tarkin and colleagues conducted a study in which gallium-68 DOTATATE (68Ga-DOTATATE) was used to detect arterial inflammation by targeting the somatostatin receptor in macrophages (76). In comparison with 18F-FDG, 68Ga-DOTATATE has preferential coronary imaging, its macrophage specificity is better, and the differentiation of high-risk vs. low coronary injury is greater. Chemokine receptor 4 (CXCR4)-targeted 68Ga-pentixaphor was used as an indicator and compared it to 18F-FDG in atherosclerotic lesions by Kircher et al. (77). Researchers showed that absorption of 68Ga-pentixaphore and 18F-FDG was poorly related when comparing lesion to lesion (*r* = 0.28; *p* < 0.01). In addition, 68Ga-Pentixaphor PET showed more lesions (*n* = 290; TBR ≥ 1.6, *p* < 0.01) and higher uptake compared to 18F-FDG PET (1.8 ± 0.5 vs. 1.4 ± 0.4, *p* < 0.01). 18F-FMISO was used in the study by Mateo et al. to indicate hypoxia in atherosclerotic plaques in rabbits. This indicator appeared to have significant uptake in the aortas of animals with atherosclerosis and also increases over time with an atherogenic diet (78). Although such investigations provide beneficial data on the molecular imaging of atherosclerotic plaques, more research is needed to obtain more detailed information on the effectiveness of these indicators.

## 9. Molecular methods for imaging inflammation

Inside the arterial wall, innate and adaptive immune reactions, provoked as a rule by clinical factors of CV risk, are the main factors determining the development of atherosclerosis and plaque rupture. Within high-risk plaques, macrophages play a crucial role in directing proinflammatory cellular signaling cascades that shape their morphology and therefore represent an attractive target for molecular imaging to track vascular inflammation (79, 80).

More than 20 years ago, it was first shown that an increased level of the 18F-FDG signal is associated with macrophages and other metabolically active inflammatory cells. Also in experimental and clinical studies, it was noted that 18F-FDG signal can be detected in vasculitis, aortic aneurysm, and atherosclerosis (81). In addition, recent studies have also shown an association between 18F-FDG uptake and hypoxia. Also, some other high-risk plaque features, such as lumen irregularity and lumen irregularity, appeared to have an association with low attenuation (82).

The capacity of plaque inflammation 18F-FDG PET in the proximal coronary beds was reported recentlyPatients were fed a low-glucose and high-lipid diet, which was aimed at overcomig the general background signal of FDG uptake by the myocardium (83). Among main limitation of the use of 18F-FDG PET for coronary arteries are cardiac and respiratory movements along with spatial resolution (3 × 3 × 3 mm voxel size) and insufficient myocardial suppression. Recently, these limitations were confirmed by the findinfg that 50% of coronary segments, especially the distal coronary bed, may not be interpreted using 18F-PD PET (84).

Atherosclerosis imaging benefited from carotid MRI, which allows to recognise plaque lipid layer, as well as plaque calcification and intra-plaque hemorrhage. Moreover, with gadolinium enhancement, MRI can reveal neovascularization/plaque permeability [K(trans)] and fibrous cap thickness/integrity. The reveal of intra-plaque hemorrhage on MRI of the carotid arteries is associated with consequent cerebrovascular events (85).

The use of ultra-small nanoparticles of superparamagnetic iron oxide (USPIO) made it possible to detect plaque inflammation specifically. USPIO are ∼30 nm diameter dextran-coated nanoparticles that have a strong T2 > T1 shortening effect, causing a signal loss in T2-weighted images [96 Macrophages uptake USPIO via phagocytosis and this is how the cellular inflammation can be detected within the tissue. Ferumoxtran-10 (Combidex in the USA, Sinerem in Europe, AMAG Pharma, and Guerbet, respectively) is the most investigated USPIO drug for atherosclerosis treatment (86). In clinical studies of carotid arteries with USPIO-MRI, asymptomatic plaques were shown to be inflamed, and the inflammation of plaques appeared to have no association with the severity of stenosis. Moreover, in symptomatic patients, fibrous caps were shown to be more inflamed and thinner. Notably, USPIO-MRI might report on dose-dependent pharmacotherapy with statins (87).

## 10. Biomechanical analysis

If atherosclerotic lesions are not uniformly distributed in the vascular system, local stimuli promote the formation and growth of plaques. Apparently, specific points in the arterial system, such as where vessels branch or curve, are more prone to developing atherosclerosis. This is because these areas experience lower shear stress, which is the frictional force of blood flow on the endothelial surface. Changes in shear stress caused by the shape of the arteries can damage the endothelium, causing inflammation and gene expression changes that impact vascular function and structure (88).

Consistently low shear stress is a critical biomechanical factor for the formation of TCFA. Structural stress due to tensile (circumferential) forces can also control the behavior of plaque, determining its capability to endure mechanical stress. The effect of structural stress on the plaques is especially significant in the presence of a weakened lid, where high mechanical stress will certainly contribute to the rupture of the plaque. Computational modeling of shear and structural stresses is possible using non-invasive and invasive visualization data (89, 90).

As a rule, to evaluate shear stress, researchers typically use CFD to simulate blood flow through 3D reconstructed vessel geometries. Anatomical data used for computer modeling of hydrodynamics may be acquired using CCTA or intravascular imaging in combination with biplane angiography. The structural stress of the plaque is influenced by numerous factors, e.g., BP, the composition, and plaque structure, the properties of the tissue material, and the lumen geometry (91, 92). The structural stress of plaque may be assessed using VH-IVUS visualization data. There are also other methods, such as FEM. The virtual computed FFR (CTFFR) may be established by biomechanical modeling, which demonstrates a positive correlation with invasive FFR (*r* = 0.7; *p* < 0.001) with a high prognostic ability (area under the curve, 0.9) to discover coronary artery stenosis >50% when assessed in clinical trials. Moreover, CTFFR can be used in the future to imitate hemodynamic changes that appear as a result of stenting when planning invasive procedures. It has been shown that this method is useful as a method of optimizing referral to invasive angiography in a prospective longitudinal study (93).

## 11. Conclusion

Unfortunately, none of the currently existing imaging methods can provide a complete and comprehensive assessment of all signs of plaque vulnerability and mechanisms of atherosclerosis development. However, advances in invasive and non-invasive imaging technologies have shown that all these methods have significant diagnostic and prognostic value. Understanding the relationship between vulnerable plaques and vulnerable patients can help not only in predicting clinical risk but also in choosing the best therapy option. In order to show how modern technological advances can be transformed from images into visualization strategies that can be widely applied in clinical settings, further large-scale clinical studies are required.

In the era of individualized medicine, the diagnostic capabilities of both modern and new methods of atherosclerosis imaging will have an impact on our care, and assistance to our patients will stimulate research of the cardiovascular system to have a broader understanding of the mechanisms of the disease and to test new treatments that are still under development. In virtue of technologies and innovations, multimodal imaging strategies with a personalized approach to each individual may be adapted to detect molecular signals with anatomical accuracy while simultaneously combining information on plaque content, blood flow patterns, and disease severity markers — therefore, shifting from a narrow focus on a plaque to a deeper understanding of the complexities underlying the basis of the pathogenesis of atherosclerosis.

**Table 1:** Overview of the main methodics of atherosclerosis imaging.

Method	Type of imaging	Invasiveness	Imaging agents	Resolution
MRI	Structural/functional	No	MNPs, Gd- nanoparticles	1–2 mm
PET	Structural/functional	No	18F, 89Zr, nanoparticles	4–5 mm
SPECT	Structural/functional	No	^18^F, ^64^Cu, ^11^C Tracers/^99m^Tc, ^123/124/125/131^I, ^111^In	4–15 mm
CT	Structural/functional	No	AuNPs, iodine-based nanoparticles	1 mm
Angiography	Structural	Yes	iodine-based	0.1 6mm
OCT	Structural	Yes	ICAM-1-targeting gold nanoshells	0.005–0.02 mm
IVUS	Structural	Yes	–	0.1 mm
OFDI	Structural	Yes	imaging agents with emission wavelengths of between 650 nm and 1,000 nm	0.01–0.02 mm
NIRF	Functional	Yes	Indocyanine green (ICG) and methylene blue	0.012 mm
